# Identifying outbreaks of sexually transmitted infection: who cares?

**DOI:** 10.1186/1471-2458-6-264

**Published:** 2006-10-24

**Authors:** Dirk Werber, Meirion R Evans, Daniel Rh Thomas

**Affiliations:** 1 Communicable Disease Surveillance Centre, National Public Health Service for Wales, Cardiff, Wales, UK; 2 European Programme for Intervention Epidemiology (EPIET)

## Abstract

**Background:**

Current routine surveillance schemes for sexually transmitted infections (STIs) in the United Kingdom (UK) are not designed for outbreak identification. Recognising STI outbreaks, therefore, depends almost entirely on the alertness of health professionals. The objective of this study was to explore health professionals' knowledge of, and attitudes towards, identification and investigation of STI outbreaks in Wales.

**Methods:**

We conducted a cross-sectional survey in Wales in June 2005, and sent a questionnaire to consultants of genitourinary medicine (GUM, n = 11), a consultant microbiologist from each laboratory (n = 14), all consultants in communicable disease control (n = 5), and to epidemiologists of the National Public Health Service (n = 4).

**Results:**

26 (76%) of 34 survey recipients responded. Of these, 17 (65%) ranked the investigation of STI outbreaks as important or very important, and 19 (73%) perceived participation in the investigation of an STI outbreak as part of their responsibility. Only six (25%) respondents had actively searched their computer system or patient records for a possible STI outbreak in the previous twelve months, and 15 (63%) had never looked for an outbreak. Of seven GUM physicians who said they had identified at least one STI outbreak, three had never informed public health authorities.

**Conclusion:**

Prompt identification and coordinated investigation of outbreaks, usually through a multidisciplinary outbreak control team, is central to the control of many infectious diseases. This does not appear to be the case for STIs, which we believe represents a lost opportunity to reduce transmission. Besides improved surveillance methods, a change in culture towards STI outbreaks is needed among health professionals in Wales.

## Background

The Health Protection Agency (HPA) ranked sexually transmitted infections (STIs), including HIV, among the greatest infectious disease threats facing the United Kingdom (UK) [1]. A network of genitourinary medicine (GUM) clinics, created after the 1916 Veneral Disease Regulations, exists to diagnose, treat and control STIs in the UK. Each year more than 1.5 million new episodes of STIs are seen in GUM clinics[1], although an increasing number are diagnosed in primary care in the UK [2].

STI outbreaks often signify a failure in routine control measures such as partner notification and treatment and warrant an effective public health response. To do so, outbreaks must first be identified. The mainstay of current surveillance of STIs in the UK is a quarterly statistical return (the KC60 form) completed by all GUM clinics [3]. This includes aggregate data on numbers of new patients seen by diagnostic category but is insufficiently detailed or timely for outbreak identification. Data are also available from voluntary laboratory reporting of confirmed STIs, but not all laboratories report, clinical information on cases is often very limited, and subtyping of isolates is not routinely available. This means that recognising possible STI outbreaks depends almost entirely on the alertness of health professionals.

The HPA has published guidelines for the investigation, management and control of acute STI outbreaks [4], indicating that "identification ... of outbreaks *can *(our italics) be by the local GUM physician, the consultant in communicable disease control, microbiologist, or regional epidemiologist". The objective of this study was to explore knowledge of, and attitudes to, STI outbreak identification and investigation among the aforementioned health professionals.

## Methods

We conducted a cross-sectional survey in Wales in June 2005. A questionnaire with a prepaid reply envelope, and a reminder letter three weeks later, was sent to all consultants in GUM (n = 11), to all consultants in communicable disease control (CCDC, n = 5), to a consultant microbiologist of each laboratory (n = 14), and to four senior epidemiologists working at the Communicable Disease Surveillance Centre of the National Public Health Service, including the three regional epidemiologists for Wales. We defined an outbreak as an unusual cluster of infections, or as more infections involving the same pathogen than normally expected. The questionnaire asked whether recipients had identified, reported (only GUM physicians and microbiologists) or investigated STI outbreaks ever, and in the previous 12 months. Furthermore, information was solicited about the perceived importance of investigating STI outbreaks, and whether GUM clinics would be allowed to share personal information with staff of the National Public Health Service in outbreak situations on a "need-to-know basis". Data were entered onto an EpiData V3.1 data base and analysed using Stata V8.2. We computed frequencies and proportions for each response category, and stratified responses according to health professional group.

## Results

26 (76%) of the 34 survey recipients completed a questionnaire (table 1). Among the respondents, 17 (65%) ranked the investigation of STI outbreaks as important or very important, and 19 (73%) perceived participation in the investigation of an STI outbreak as part of their responsibility (table 1). Six (25%) of 24 respondents had actively searched their computer system or patient records for a possible STI outbreak at least once in the last twelve months; 16 (63%) had never looked for an outbreak. Of seven GUM physicians who said they had identified at least one STI outbreak before, four had ever informed the public health authorities. The three non-reporters considered that contact tracing conducted by staff of the GUM clinic was sufficient to control the outbreak. During the five years prior to this survey, only one STI outbreak in Wales led to the setting up of a multi-disciplinary outbreak control team resulting in detailed contact investigations and proactive control measures. 12 (46%) of 26 respondents believed that GUM clinics should not, or are not allowed to, share personal information in an STI outbreak on a need-to-know basis (table 2).

## Discussion

This study suggests that few STI outbreaks are currently recognised in Wales because few health professionals are trying to identify them. Even fewer STI outbreaks get reported to public health authorities and result in a multidisciplinary response. Furthermore, substantial uncertainty exists about confidentiality of patient information in STI outbreak situations. Since surveillance schemes for STIs are similar throughout the UK, it is conceivable that a comparable situation exists in areas of the other UK countries.

To our knowledge, no study has yet addressed the issue of alertness of health professionals to identify STI outbreaks. Of interest, identifying and investigating STI outbreaks is not explicitly mentioned as an objective of STI surveillance schemes in the UK [3].

The control of unrecognised outbreaks and, according to this survey, of some of the recognised outbreaks, is by routine partner notification and treatment only. These interventions, however, are limited in their effectiveness [5]. For example, anonymity of sexual partners, particularly for men who have sex with men [5], and delays in partner notification and treatment [6] (e.g., because of waiting times at GUM services for initial consultation [7], or delayed notification of partners by the index patient) are impediments for timely interruption of onward transmission. A systematic outbreak investigation, usually by a multidisciplinary outbreak control team, can facilitate a multifaceted response to the outbreak that is adapted to the size and circumstances of the incident. This might include, for example, active case finding by prompting awareness among providers and outreach workers, and selective screening of patients with similar or milder clinical presentations. A more network-informed approach could identify central persons in the sexual network (likely to be core group members), and extend case finding efforts to nonsexual contacts of cases [8]. Together with descriptive epidemiology, approaches like this could lead to more complete ascertainment and timelier identification of outbreak cases and their contacts, and a more accurate description of the population at risk. In consequence, earlier treatment and education of a larger number of outbreak-related cases could reduce spread of infection and promote the number of persons that modify their risk-behaviour. Furthermore, the use of analytical epidemiological studies may identify risk factors that allow for a more targeted intervention approach [9].

Although patient confidentiality is a central tenet of GUM practice, the law allows sharing of patient information with other health professionals in the interests of controlling spread[4]. It is noteworthy that only half of the survey respondents thought this was true and should be practiced in STI outbreaks. This indicates not only difficulties for an effective multidisciplinary response to STI outbreaks in many instances, but also that a common conceptual framework of how to cooperate with public health authorities in STI outbreaks does not currently exist in Wales. Clearer definition of roles in STI outbreak identification and control, particularly on the local level, are needed. Setting up of regular sexual health liaison meetings involving GUM physicians, public health officials (e.g., CCDC and public health nurses), and representatives of Local Health Boards may increase mutual understanding and thereby help in defining the roles of these groups – not only in outbreak situations. Furthermore, improved surveillance methods based on individual data with a more timely data flow to regional epidemiologists or to local public health authorities (i.e., the Health Protection Team in the UK) would enable these professionals to identify increases in notification data indicative of STI outbreaks.

This study targeted health professionals across the whole of Wales that might potentially identify STI outbreaks. Yet, the number of survey recipients was small. Extending the study to other UK countries would provide the statistical power to compare among health professions, and possibly reveal regional differences in practice in the UK. This would help in identifying areas where, in particular, STI surveillance efforts need to be strengthened.

## Conclusion

Prompt identification and coordinated investigation of outbreaks is central to the control of many infectious diseases. This does not appear to be the case for STIs, which we believe represents a lost opportunity to reduce or even stop transmission. To effectively control STI outbreaks in Wales, and possibly in other countries of the UK, improved surveillance methods based on timely and detailed ascertainment of individual cases are needed, as well as convincing health professionals of the importance of identifying and investigating STI outbreaks.

## Competing interests

The author(s) declare that they have no competing interests.

## Authors' contributions

DW designed the questionnaire, collected and analysed the data, and wrote the manuscript. MRE helped in designing the study and revised the manuscript. DRhT supervised the study, co-wrote and revised the article. All authors read and approved the final manuscript.

## Pre-publication history

The pre-publication history for this paper can be accessed here:



## Figures and Tables

**Table 1:** Attitudes of stakeholders in STI surveillance schemes in Wales towards perceived importance of and responsibility for investigating STI outbreaks – contrasted by efforts of identifying these outbreaks.

Health Profession	Participation		Investigating STI outbreaks is important*		Am responsible for participating in STI outbreak investigation		Actively looked for STI outbreaks in last 12 months	
	n/N	(%)	n/N	(%)	n/N	(%)	n/N	(%)
Microbiologist	8/14	(57)	1/8	(13)	5/8	(57)	1/7	(14)
GUM ^† ^Consultant	9/11	(82)	9/9	(100)	7/9	(78)	3/9	(33)
CCDC ^†^	5/5	(100)	4/5	(80)	4/5	(80)	0/4	(0)
CDSC-Epidemiologist^†^	4/4	(100)	3/4	(75)	4/4	(100)	2/4	(50)

Total	26/34	(76)	17/26	(65)	19/26	(73)	6/24	(25)

* Proportion of participants who ranked investigation of STI outbreaks as important or very important as compared to: not important, or slightly important, or moderately important

^† ^GUM = Genitourinary Medicine, CCDC = Consultant in Communicable Disease Control,

CDSC = Communicable Disease Surveillance Centre

**Table 2:** Are staff of the GUM clinic allowed to share personal information during STI outbreaks with staff of the National Public Health Service on a "need-to-know" basis?.

Health Profession	Yes		Yes, but should be discouraged*		No		Don't know	
	n	(%)	n	(%)	n	(%)	n	(%)
Microbiologist (n = 8)	3	(38)	2	(25)	2	(25)	1	(12)
GUM Consultant (n = 9)	5	(56)	2	(22)	2	(22)	0	(0)
CCDC (n = 5)	3	(60)	0	(0)	2	(40)	0	(0)
CDSC-Epidemiologist^† ^(n = 4)	2	(50)	1	(25)	1	(25)	0	(0)

Total	13	(50)	5	(19)	7	(27)	1	(4)

* because patients may regard this as a confidentiality breach

## References

[B1] The UK Collaborative Group for HIV and STI Surveillance (2004). Focus on Prevention. HIV and other Sexually Transmitted Infections in the United Kingdom in 2003. London: Health Protection Agency Centre for Infections November 2004.

[B2] Cassell JA, Mercer CH, Sutcliffe L, Petersen I, Islam A, Brook MG, Ross JD, Kinghorn GR, Simms I, Hughes G, Majeed A, Stephenson JM, Johnson AM, Hayward AC (2006). Trends in sexually transmitted infections in general practice 1990–2000: population based study using data from the UK general practice research database. BMJ.

[B3] Hughes G, Catchpole M (1998). Surveillance of sexually transmitted infections in England and Wales. Eurosurveillance weekly.

[B4] HPA (2002). Guidelines for managing outbreaks of sexually transmitted infections at a local, regional or district level. http://www.hpa.org.uk/infections/topics_az/hiv_and_sti/guidelines/sti_outbreakplan.pdf.

[B5] Hogben M, Paffel J, Broussard D, Wolf W, Kenney K, Rubin S, George D, Samoff E (2005). Syphilis partner notification with men who have sex with men: a review and commentary. Sex Transm Dis.

[B6] White PJ, Ward H, Cassell JA, Mercer CH, Garnett GP (2005). Vicious and virtuous circles in the dynamics of infectious disease and the provision of health care: gonorrhea in Britain as an example. J Infect Dis.

[B7] Griffiths V, Ahmed-Jushuf I, Cassell JA (2004). Understanding access to genitourinary medicine services. Int J STD AIDS.

[B8] Rothenberg R, Kimbrough L, Lewis-Hardy R, Heath B, Williams OC, Tambe P, Johnson D, Schrader M (2000). Social network methods for endemic foci of syphilis: a pilot project. Sex Transm Dis.

[B9] De P, Singh AE, Wong T, Yacoub W (2003). Outbreak of Neisseria gonorrhoeae in Northern Alberta, Canada. Sex Transm Dis.

